# Isolation of microorganisms involved in reduction of crystalline iron(III) oxides in natural environments

**DOI:** 10.3389/fmicb.2015.00386

**Published:** 2015-05-05

**Authors:** Tomoyuki Hori, Tomo Aoyagi, Hideomi Itoh, Takashi Narihiro, Azusa Oikawa, Kiyofumi Suzuki, Atsushi Ogata, Michael W. Friedrich, Ralf Conrad, Yoichi Kamagata

**Affiliations:** ^1^Environmental Management Research Institute, National Institute of Advanced Industrial Science and TechnologyTsukuba, Japan; ^2^Bioproduction Research Institute, National Institute of Advanced Industrial Science and TechnologySapporo, Japan; ^3^Bioproduction Research Institute, National Institute of Advanced Industrial Science and TechnologyTsukuba, Japan; ^4^Methane Hydrate Research & Development Division, Japan Oil, Gas and Metals National CorporationChiba, Japan; ^5^Microbial Ecophysiology Group, Faculty of Biology/Chemistry and MARUM, University of BremenBremen, Germany; ^6^Max Planck Institute for Terrestrial MicrobiologyMarburg, Germany

**Keywords:** isolation, iron-reducing bacteria, crystalline iron(III) oxide, *Geobacter*, high-throughput sequencing

## Abstract

Reduction of crystalline Fe(III) oxides is one of the most important electron sinks for organic compound oxidation in natural environments. Yet the limited number of isolates makes it difficult to understand the physiology and ecological impact of the microorganisms involved. Here, two-stage cultivation was implemented to selectively enrich and isolate crystalline iron(III) oxide reducing microorganisms in soils and sediments. Firstly, iron reducers were enriched and other untargeted eutrophs were depleted by 2-years successive culture on a crystalline ferric iron oxide (i.e., goethite, lepidocrocite, hematite, or magnetite) as electron acceptor. Fifty-eight out of 136 incubation conditions allowed the continued existence of microorganisms as confirmed by PCR amplification. High-throughput Illumina sequencing and clone library analysis based on 16S rRNA genes revealed that the enrichment cultures on each of the ferric iron oxides contained bacteria belonging to the Deltaproteobacteria (mainly Geobacteraceae), followed by Firmicutes and Chloroflexi, which also comprised most of the operational taxonomic units (OTUs) identified. Venn diagrams indicated that the core OTUs enriched with all of the iron oxides were dominant in the Geobacteraceae while each type of iron oxides supplemented selectively enriched specific OTUs in the other phylogenetic groups. Secondly, 38 enrichment cultures including novel microorganisms were transferred to soluble-iron(III) containing media in order to stimulate the proliferation of the enriched iron reducers. Through extinction dilution-culture and single colony isolation, six strains within the Deltaproteobacteria were finally obtained; five strains belonged to the genus *Geobacter* and one strain to *Pelobacter*. The 16S rRNA genes of these isolates were 94.8–98.1% identical in sequence to cultured relatives. All the isolates were able to grow on acetate and ferric iron but their physiological characteristics differed considerably in terms of growth rate. Thus, the novel strategy allowed to enrich and isolate novel iron(III) reducers that were able to thrive by reducing crystalline ferric iron oxides.

## Introduction

Iron (Fe) is the fourth most common element in the Earth’s crust, and the vast majority is distributed as iron minerals in natural environments such as soils and sediments (Stumm and Sulzberger, 1992; Davison, 1993; Weber et al., 2006). Ferric iron [Fe(III)] and ferrous iron [Fe(II)] are the main redox states in the environments. The microbially mediated and abiotic redox cycling of iron play pivotal roles in global biogeochemistry, e.g., the degradation and preservation of organic matters, and the fate of contaminants and nutrients for living organism (Borch et al., 2010; Lalonde et al., 2012; Melton et al., 2014). Furthermore, microbial Fe(III) reduction is one of the most significant electron sinks for the oxidation of organic compounds under anoxic conditions prevailing in natural ecosystems (Yao et al., 1999; Lovley et al., 2004; Hori et al., 2010; Braunschweig et al., 2013). Thereby it exerts strong influences on global climate change as a competitor to methanogenesis that is the dominant terminal reduction process to produce the greenhouse effect gas methane (Conrad, 1996; Yvon-Durocher et al., 2014).

Crystalline iron oxides (or hydroxides), such as goethite (α-FeOOH), lepidocrocite (γ-FeOOH), hematite (α-Fe_2_O_3_), and magnetite (Fe_3_O_4_), are known to be more abundant than amorphous iron species, e.g., ferrihydrite, in soils and subsurface sediments (Roden and Urrutia, 2002; Cornell and Schwertmann, 2004; Weber et al., 2006). Whereas ferrihydrite has been used as electron acceptor for cultivating ferric iron reducers due to its easier accessibility for microorganisms to use (Treude et al., 2003; Lovley et al., 2004), the reduction of crystalline iron oxides had been considered either undetectable or quite small in extent (Lovley and Phillips, 1986a, 1987, 1988; Qu et al., 2004; Kukkadapu et al., 2006). However, previous studies have suggested that iron reducers could thrive at the thermodynamic limits by utilizing structural or crystalline iron(III) oxides as electron acceptors (Kostka and Nealson, 1995; Kostka et al., 1996; Fredrickson et al., 1998; Kukkadapu et al., 2001). In addition, substitution by aluminum, commonly found co-precipitated with Fe(III) oxides, has resulted in declined microbial reduction of the amorphous ferrihydrite whereas it has not impacted the reduction of the crystalline goethite and lepidocrocite (Ekstrom et al., 2010), giving rise to the possibility that reduction of crystalline iron oxides is commonly occurring in natural environments (van der Zee et al., 2003). Recently, in anoxic paddy soils, novel iron(III) reducers assimilating acetate, albeit at low rates, in the presence of goethite have been identified by means of stable isotope probing (SIP; Hori et al., 2010; Ding et al., 2015). Yet the diversity of the microorganisms involved in reduction of crystallized ferric irons and the mechanisms underlying the reduction process are largely unknown. A limited number of isolates makes it difficult to understand the physiology and ecological impact of the microorganisms. Lentini et al. (2012) have enriched iron(III)-reducing bacterial communities with iron-oxide minerals but not attempted to isolate the predominant microorganisms as pure cultures.

Several isolation techniques and cultivation strategies have been developed, in order to get access to hitherto uncultured microorganisms such as slow growers, oligotrophs, and fastidious microorganisms (Puspita et al., 2012; Narihiro and Kamagata, 2013; Prakash et al., 2013). We propose herein a new two-stage cultivation method for selectively isolating the microorganisms potentially capable of reducing iron mineral phases with high crystallinity. First, untargeted eutrophs were depleted by long-term successive culturing on a crystalline iron oxide (i.e., goethite, lepidocrocite, hematite, or magnetite) as electron acceptor and acetate as electron donor. Fe(III) reducers typically adhere to the iron oxide surface probably facilitating electron transfer (Lower et al., 2001; Rosso et al., 2003). Thus, they most likely survive on the solid–liquid interface for prolonged periods. After being highly enriched, proliferation of the targeted iron reducers was enhanced during a second cultivation with energetically more favorable hydrous iron(III) media. Throughout the two-stage cultivation processes, a variety of molecular ecological tools [i.e., high-throughput Illumina sequencing, terminal restriction fragment length polymorphism (T-RFLP), and clone library analyses of 16S rRNA gene amplicons] were applied to monitor microbial community structures and to facilitate the isolation of phylogenetically novel microorganisms involved in reduction of crystalline iron oxides.

## Materials and Methods

### First Cultivation Stage: Enrichment Culture with Crystalline Iron Oxides

**FIGURE 1:**
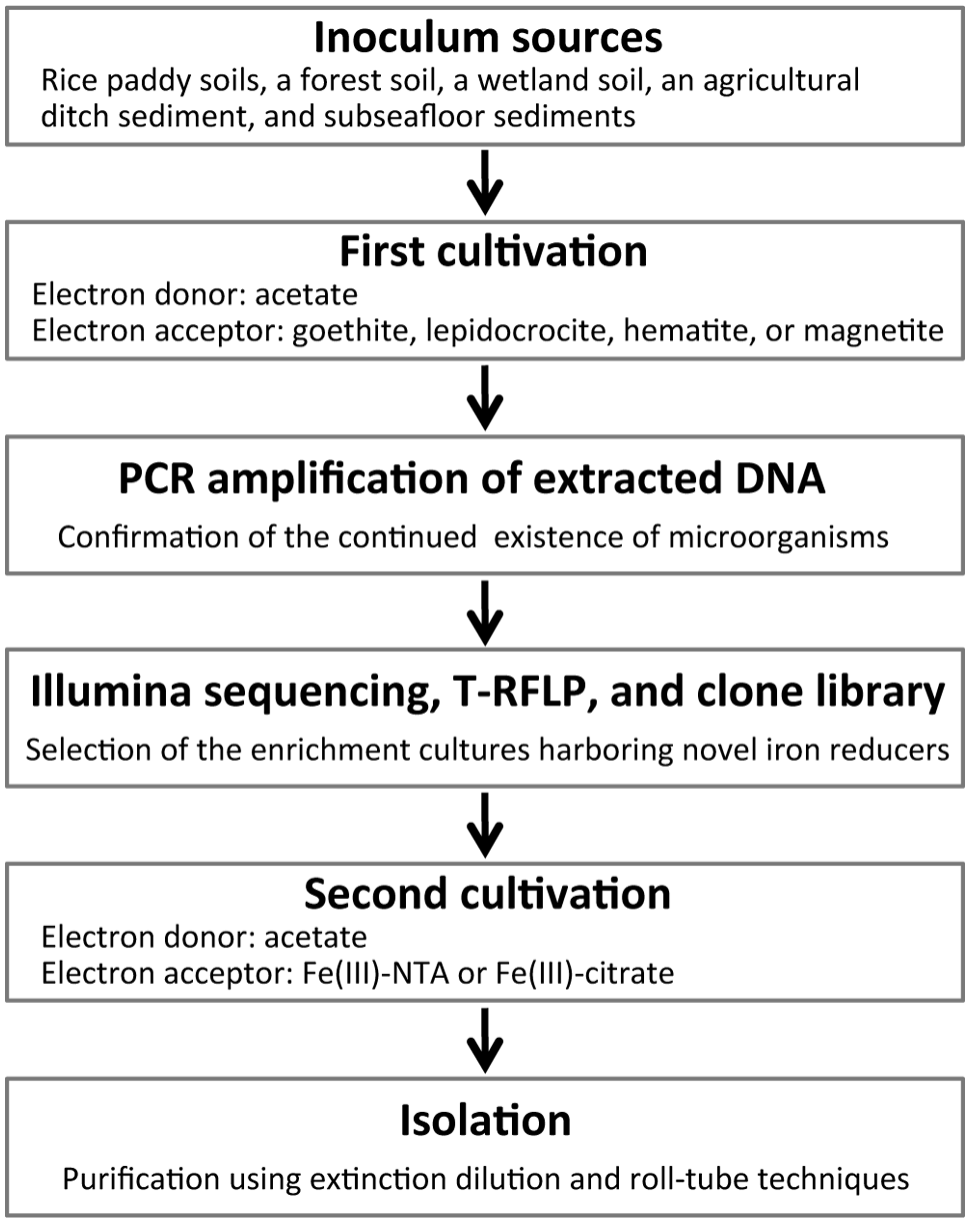
**A flow chart of the two-stage cultivation method proposed in this study**.

Schematic overview of the two-stage cultivation method proposed in this study is shown in **Figure 1**. As inoculum sources of microorganisms, soil and sediment samples including rice paddy soils, a forest soil, a wetland soil, an agricultural ditch sediment, and subseafloor sediments were collected. Rice paddy soils were obtained from three different places (i.e., rice fields in Hokkaido and Okayama, Japan, and the Italian Rice Research Institute in Vercelli, Italy). These sampling sites were previously described in detail (Schütz et al., 1989; Narihiro et al., 2011) except that in Okayama (34°64^′^N, 133°84^′^E). Collection sites of forest and wetland soils were mentioned elsewhere (Narihiro et al., 2011, 2014). The ditch sediment was obtained from an agricultural stream near the above mentioned wetland (Narihiro et al., 2011). Subseafloor sediments were sampled from a drilling site (34°16^′^N, 137°57^′^E) of the Nankai Trough (Tsuji et al., 2004). The soils and sediments were mixed with 10 mM phosphate buffer. Aliquots (2 ml) of homogenized slurries were anaerobically inoculated to two types of freshwater basal media (18 ml) in 50-ml glass serum vials, and then sealed with butyl rubber septa. The basal media were either the modified Widdel medium (Kanno et al., 2013) or the DSMZ medium 579. The modified Widdel medium (pH 7.0) consisted of (in grams per liter) NH_4_Cl, 0.535; KH_2_PO_4_, 0.136; MgCl_2_∙6H_2_O, 0.204; CaCl_2_∙2H_2_O, 0.147; NaHCO_3_, 2.52; as well as a selenium and tungsten solution, 1 ml; a trace element solution, 1 ml; and a vitamin solution, 2 ml. Composition of the modified DSMZ-579 medium (pH 7.0) was (per liter): NaHCO_3_, 2.5 g; NH_4_Cl, 1.5 g; NaH_2_PO_4_, 0.6 g; KCl, 0.1 g; Na_2_WO_4_∙2H_2_O, 0.25 mg; in addition to a trace element solution, 10 ml; and a vitamin solution, 10 ml. A crystalline iron(III) oxide (or hydroxide), i.e., goethite (α-FeOOH; particle size, ca. 10 μm; Alfa Aesar), lepidocrocite (γ-FeOOH; particle size, <250 μm; Alfa Aesar), hematite (α-Fe_2_O_3_; particle size, ca. 10 μm; Wako), or magnetite (Fe_3_O_4_; particle size, 20–30 nm; Alfa Aesar), was supplemented as an electron acceptor at a final concentration of 20 mM (for goethite and lepidocrocite) or 10 mM (for hematite and magnetite). The media were amended with acetate as electron donor at a concentration of 20 mM. For inhibiting growth of methanogens, 2-bromoethanesulfonate (BES, 20 mM) was used (Zhou et al., 2011). A total of 136 incubation conditions were generated from combinations among the sampling sites, basal media, electron acceptors, and presence or absence of BES. After flushing the headspace of the incubation vials with N_2_/CO_2_ (80:20), the enrichment cultures (volume of 20 ml) were kept in the dark at 25°C. When the color of media changed, i.e., the added iron oxide was possibly reduced, 10% of the enrichment cultures including solid-phase iron oxides were transferred to fresh media. During the incubation for ∼2 years, subculturing was carried out two or three times depending on the time of color change.

### Fine-Scale Analyses of the Community Structures in Microbial Enrichment Cultures

Nucleic acids were extracted from the twice- or thrice-transferred enrichment cultures using a direct lysis protocol with bead beating (Noll et al., 2005). RNA was removed from total DNA extracts by RNase (Type II-A; Sigma-Aldrich) digestion. The resulting DNA extract was used as a template for bacterial 16S rRNA genes targeting PCR with primers B27f and B907r (Hori et al., 2007) and GoTaq Flexi DNA polymerase (Promega) for tracking enrichment of microorganisms during the first cultivation. Thermal conditions of PCR were as follows: an initial denaturation at 94°C for 3 min, and then 30–45 cycles of denaturation at 94°C for 30 s, annealing at 52°C for 45 s, extension at 72°C for 90 s, and a final extension step at 72°C for 5 min. The amplicons were checked by electrophoresis on 1% agarose gels before being subjected to molecular analyses, i.e., Illumina sequencing, T-RFLP, and clone library analyses.

For Illumina sequencing, experimental steps and data analyses were described in detail elsewhere (Aoyagi et al., 2015). Shortly, PCR was carried out with a Q5 High-Fidelity DNA polymerase (New England Biolabs) using the universal primer set 515F/806R (Caporaso et al., 2012). These primers contain Illumina adaptors, and the reverse one was encoded with 6-bp barcodes. The PCR thermal profile was described previously (Hori et al., 2014), except that a total of 30–40 cycles was employed. The PCR amplicon was purified firstly with an AMPure XP kit (Beckman Coulter), and then subjected to second purification with a QIAquick gel extraction kit (QIAGEN). The generated DNA libraries and an initial control (PhiX; Illumina) were sequenced with a 300-cycle MiSeq Reagent kit (Illumina) on a MiSeq sequencer (Illumina). PhiX, low-quality (Q < 30), and chimeric sequences were removed and paired-end sequences (≥Q30) were assembled as previously described (Itoh et al., 2014). A total of 2,131,707 sequences (an average of 36,754 sequences per library) were characterized phylogenetically using the QIIME software package, version 1.7.0 (Caporaso et al., 2010). After taxonomic assignment, a self-written script removed archaeal sequences in the libraries. Operational taxonomic units (OTUs) were defined using a cut-off of 97% sequence identity. Alpha diversity indices (i.e., Chao1, Shannon, and Simpson reciprocal) and beta diversity [i.e., the weighted UniFrac distances for principal coordinate analysis (PCoA)] were calculated using QIIME (Caporaso et al., 2010). By using a self-written script, OTUs specifically found in libraries with one iron oxide and the others shared in libraries with multiple other iron oxides were differentiated to construct Venn diagrams.

With respect to T-RFLP analysis, PCR was performed with the primer set B27f/B907r under thermal conditions as described above, except that the forward primer was fluorescently labeled (Beckmann Dye D4; Sigma-Aldrich) and a total of 25–30 PCR cycles were employed. Sample preparation, electrophoresis, and data analyses were performed as described previously (Akuzawa et al., 2011). Briefly, the PCR product was purified with a QIAquick PCR purification kit (QIAGEN), and 200 ng of the amplicon was digested with the restriction enzyme *MspI* (New England Biolabs). Prior to applying electrophoresis, the digests were added to a loading solution (Beckman Coulter) containing a GenomeLab DNA size standard kit – 600 (Beckman Coulter). T-RFs were size-separated by capillary electrophoresis with a GenomeLab GeXP (Beckman Coulter). T-RFLP profile was determined on the basis of the peak area and height with a CEQ8000 Genetic Analysis System (Beckman Coulter). Principal component analysis (PCA) based on the size and relative abundance of T-RFs was performed using the software JMP, version 5.1 (SAS Institute).

For clone library analysis, bacterial 16S rRNA genes were amplified by PCR with the primer set B27f/B907r under the same thermal conditions for T-RFLP. The PCR amplicon was purified using a QIAquick PCR purification kit (QIAGEN) and then ligated into the plasmid vector pGEM-T Easy (Promega). *Escherichia coli* JM109 supercompetent cells (Nippon Gene) were transformed with the generated plasmid according to the manufacturer’s instructions. A total of 525 clones were randomly selected, for which approximately 10 clones were retrieved from each library. The extracted DNA portion was sequenced using a BigDye Terminator v3.1 Cycle Sequencing kit and a 3130xl Genetic Analyzer (Applied Biosystems). The 16S rRNA gene sequences obtained were compared with those in the nucleotide sequence database by using the BLAST program. Furthermore, phylogenetic trees of the obtained partial sequences and nearly full-length reference sequences were constructed using the ARB software^1^ (Ludwig et al., 2004) as described previously (Hori et al., 2007).

### Second Cultivation Stage: Subculture and Isolation of Iron Reducers with Soluble Ferric Iron

Microbial enrichment cultures in which phylogenetically novel microorganisms dominated were transferred to energetically more favorable soluble-iron(III) media. In this context, the employed electron acceptor was changed from a crystalline iron(III) oxide to a soluble Fe(III) species, i.e., ferric nitriloacetic acid [Fe(III)-NTA; 10 mM] or ferric citrate (30 mM). Carbon source was acetate at concentrations of 10–20 mM. The basal medium (the Widdel medium or the DSMZ-579 medium) from the first cultivation was used. Extinction dilution and Hungate roll-tube technique (Hungate, 1969) were implemented to isolate novel microorganisms. In the roll-tube method, soluble iron(III) media were solidified with 2% Noble agar. The definite isolation of microorganisms as pure cultures was confirmed by microscopic observation and molecular analyses, i.e., Illumina sequencing and T-RFLP of 16S rRNA genes.

### Phylogenetic and Physiological Analyses of Isolated Microorganisms

For phylogenetic analysis of isolates, nucleic acids were extracted from pure cultures as described above and nearly full length of 16S rRNA gene was amplified by PCR with a GoTaq Flexi DNA polymerase (Promega) using the primer set B27f/B1525r (Lane, 1991). The PCR thermal program mentioned above was slightly modified: extension time was set longer (i.e., 2 min) in each cycle of the PCR program. The PCR product was subjected to cloning and transformation as described for clone library analysis. The DNA segment was sequenced with a CEQ Dye Terminator Cycle Sequencing (DTCS) Quick Start kit (Beckman Coulter) and a GenomeLab GeXP (Beckman Coulter). The obtained sequences of 16S rRNA genes were aligned with the Clustal_X software, version 2.0.1 (Larkin et al., 2007). Phylogenetic trees were constructed by the neighbor-joining and maximum likelihood methods using the software MEGA, version 5.2 (Tamura et al., 2011). Similar topologies of the trees with different algorithms were confirmed. Robustness of the branch clustering was assessed by bootstrap values on the basis of 1,000 replications.

For physiological characterization of isolates, Fe(III) reduction, acetate degradation, cell growth, and protein synthesis were determined during the axenic incubation, in which Fe(III)-NTA (10 mM) and acetate were used as electron acceptor and donor, respectively. The basal medium was either the modified Widdel medium (Kanno et al., 2013) or the DSMZ-579 medium to be the same with that used for the second cultivation. Total and ferrous irons were determined as described earlier (Achtnich et al., 1995). Briefly, 0.5 ml of the incubation culture were mixed with HCl at a final concentration of 0.5 N, and incubated for 14 to 16 h. Fe(II) and hydroxylamine reducible Fe(III) were determined by the ferrozine method. Concentration of acetate was measured by a high-pressure liquid chromatograph (Alliance e26951; Waters) equipped with an RSpak KC-811 column (Shodex) and a photodiode array (2998; Waters). Quantitative PCR (qPCR) of 16S rRNA genes was used to monitor cell growth of isolates and carried out with a GoTaq qPCR Master Mix using the primer set 515F/806R. The thermal program was based on the manufacturer’s instruction. The total copy numbers at the beginning of the axenic incubation ranged from 2.6 × 10^6^ copies ml^-1^ to 3.1 × 10^7^ copies ml^-1^. To create a standard curve for quantification, dilution series (10^5^ to 10^11^ copies μl^-1^) of 16S rRNA genes from *E. coli* cells was utilized. A melting curve analysis was performed between 60 and 95°C to check the absolute amplification of a genomic DNA from an isolate. Total protein was obtained from the incubated cells that were broken up using an ultrasonic disintegrator (UD-201; TOMY) and its concentration was determined on the basis of the Bradford dye-binding method with a Quick Start Bradford Protein Assay (Bio-Rad) and a microplate reader (SH-9000Lab; Corona Electric).

### Nucleotide Sequence Accession Numbers

Nucleotide sequences retrieved from the clone-library analysis of microbial enrichment cultures and the phylogenetic analysis of isolates were deposited in the DDBJ nucleotide sequence database^2^ under accession numbers AB999079 to AB999603 (525 clones) and LC008327 to LC008332 (six isolates), and those obtained from Illumina sequencing analysis of microbial enrichment cultures were deposited in the MG-RAST database^3^ (Meyer et al., 2008) as a project of “Microbial communities enriched with acetate and crystalline iron(III) oxides in 2014” under the accession IDs 4589479.3–4589493.3, 4589495.3–4589536.3, and 4610839.3 (58 libraries).

## Results and Discussion

### Microbial Enrichment Cultures on Crystalline Iron Oxides using Inocula from a Variety of Soils and Sediments

The energy obtained for microbial growth was apparently severely limited during the first cultivation, as it took a half year or longer to observe ferric iron reduction, i.e., the color change of the incubation media. Less changes were visible during the incubation of subseafloor sediments compared to those of the other soils and sediments, and thus subculturing was done only two times for the subseafloor incubation while three times for the other incubations. The structural changes in iron(III) minerals most likely occurred during the incubation process. At the end of the long-term incubation, the continued existence of microorganisms was confirmed by PCR amplification with genomic DNAs extracted from the enrichment cultures. Positive results were obtained in 58 out of the total 136 incubation conditions (**Table 1**).

**Table 1:** Microbial diversity indices (i.e., Chao1, Shannon, and Simpson reciprocal) determined based on Illumina sequencing data.

Inoculum source	Library ID	Basal medium^b^	BES	Iron(III) oxide	Alpha diversity index^a^		
					Chao1^c^	Shannon^c^	1/Simpson^c^
**Rice paddy soil^**d**^**
Italy	e05	W	+	Goethite	860 (±83)	6.29 (±0.04)	35.3 (±1.1)
Italy	e11	W	-	Goethite	663 (±45 )	5.58 (±0.03)	21.8 (±0.4)
Italy	e50	D	+	Goethite	1626 (±271)	4.72 (±0.04)	8.6 (±0.2)
Italy	e35	D	-	Goethite	417 (±111)	3.30 (±0.04)	5.2 (±0.2)
Okayama	e27	W	+	Goethite	1369 (±141)	5.29 (±0.03)	11.8 (±0.2)
Okayama	e14	W	-	Goethite	1731 (±106)	6.82 (±0.04)	49.8 (±1.1)
Okayama	e39	D	-	Goethite	1297 (±121)	4.60 (±0.04)	8.2 (±0.1)
Hokkaido	e18	W	-	Goethite	1654 (±246)	5.86 (±0.05)	22 (±0.5)
Italy	e06	W	+	Lepidocrocite	994 (±120)	5.40 (±0.05)	15.1 (±0.4)
Italy	e10	W	-	Lepidocrocite	1388 (±175)	5.32 (±0.04)	11.7 (±0.3)
Italy	e51	D	+	Lepidocrocite	816 (±84)	3.81 (±0.05)	5.2 (±0.1)
Italy	e36	D	-	Lepidocrocite	548 (±65)	3.31 (±0.04)	5.2 (±0.1)
Okayama	e28	W	+	Lepidocrocite	847 (±131)	4.45 (±0.03)	7.1 (±0.2)
Okayama	e15	W	-	Lepidocrocite	1011 (±129)	5.77 (±0.02)	21 (±0.5)
Okayama	e54	D	+	Lepidocrocite	476 (±92)	2.42 (±0.03)	2.9 (±0.04)
Okayama	e40	D	-	Lepidocrocite	1153 (±188)	4.29 (±0.03)	8.8 (±0.3)
Hokkaido	e31	W	+	Lepidocrocite	965 (±140)	3.54 (±0,04)	3.3 (±0.1)
Hokkaido	e19	W	-	Lepidocrocite	1736 (±192)	4.61 (±0.06)	7.8 (±0.2)
Hokkaido	e56	D	+	Lepidocrocite	902 (±171)	3.22 (±0.03)	5 (±0.1)
Hokkaido	e43	D	-	Lepidocrocite	788 (±131)	2.49 (±0.04)	2.7 (±0.04)
Italy	e09	W	-	Hematite	922 (±114)	4.52 (±0.03)	7.5 (±0.2)
Italy	e49	D	+	Hematite	541 (±101)	3.85 (±0.03)	7.5 (±0.1)
Okayama	e13	W	-	Hematite	1883 (±214)	6.09 (±0.07)	17.7 (±0.9)
Okayama	e53	D	+	Hematite	672 (±151)	3.17 (±0.04)	4.5 (±0.1)
Okayama	e38	D	-	Hematite	627 (±115)	3.12 (±0.05)	3.2 (±0.1)
Hokkaido	e30	W	+	Hematite	961 (±90)	4.67 (±0.04)	9 (±0.2)
Hokkaido	e17	W	-	Hematite	951 (±130)	4.97 (±0.05)	14 (±0.2)
Hokkaido	e42	D	-	Hematite	781 (±124)	2.07 (±0,05)	1.8 (±0.03)
Italy	e07	W	+	Magnetite	547 (±112)	3.06 (±0.06)	3.2 (±0.1)
Italy	e08	W	-	Magnetite	1039 (±117)	4.85 (±0.04)	7.6 (±0.3)
Italy	e52	D	+	Magnetite	325 (±90)	3.26 (±0.04)	5.5 (±0.1)
Italy	e37	D	-	Magnetite	533 (±66)	4.43 (±0.06)	10 (±0.3)
Okayama	e29	W	+	Magnetite	719 (±69)	4.44 (±0.05)	7.4 (±0.2)
Okayama	e16	W	-	Magnetite	1414 (±121)	6.16 (±0.01)	28.2 (±0.3)
Okayama	e55	D	+	Magnetite	682 (±130)	3.88 (±0.06)	5.6 (±0.2)
Okayama	e41	D	-	Magnetite	718 (±45)	4.24 (±0.04)	5.3 (±0.2)
Hokkaido	e32	W	+	Magnetite	673 (±67)	4.47 (±0.04)	8.7 (±0.2)
Hokkaido	e20	W	-	Magnetite	891 (±131)	4.85 (±0.06)	7.9 (±0.3)
Hokkaido	e57	D	+	Magnetite	374 (±45)	3.20 (±0.05)	4.7 (±0.1)
Hokkaido	e44	D	-	Magnetite	588 (±153)	4.51 (±0.02)	12.2 (±0.2)
**Agricultural ditch sediment**
	e24	W	-	Goethite	612 (±52)	4.61 (±0.05)	9.2 (±0.2)
	e46	D	-	Goethite	385 (±81)	1.23 (±0.05)	1.4 (±0.02)
	e33	W	+	Lepidocrocite	400 (±69)	2.09 (±0.04)	2.1 (±0.02)
	e25	W	-	Lepidocrocite	405 (±63)	4.15 (±0.03)	9.3 (±0.3)
	e45	D	-	Hematite	504 (±78)	1.67 (±0.06)	1.6 (±0.03)
	e34	W	+	Magnetite	341 (±60)	3.98 (±0.05)	7.3 (±0.2)
	e26	W	-	Magnetite	1183 (±82)	5.51 (±0.06)	10.6 (±0.5)
	e59	D	+	Magnetite	263 (±40)	3.40 (±)0.04	5.4 (±0.1)
	e48	D	-	Magnetite	310 (±45)	3.35 (±0.03)	5.3 (±0.1)
**Subseafloor sediment**
	e65	W	-	Goethite	1195 (±145)	4.53 (±0.06)	7.3 (±0.2)
	e66	D	-	Goethite	333 (±52)	1.91 (±0.04)	1.9 (±0.02)
	e62	W	-	Goethite	1445 (±124)	5.31 (±0.05)	12.1 (±0.3)
	e60	W	-	Goethite	961 (±102)	4.67 (±0.04)	12.2 (±0.2)
	e61	W	-	Lepidocrocite	608 (±107)	3.70 (±0.04)	7.3 (±0.1)
	e64	W	-	Hematite	332 (±56)	1.81 (±0.05)	1.7 (±0.03)
**Wetland soil**
	e21	W	-	Lepidocrocite	766 (±176)	3.12 (±0.03)	4.9 (±0.1)
	e22	W	-	Magnetite	477 (±73)	2.23 (±0.05)	2.6 (±0.04)
**Forest soil**
	e01	W	-	Magnetite	832 (±105)	3.42 (±0.02)	4.4 (±0.1)

^a^Each index was calculated based on equal numbers of sequences (*n* = 3,544) sub-sampled from the bacterial libraries.

^b^The basal medium utilized is indicated by W or D (W: the modified Widdel medium, D: the modified DSMZ 579 medium).

^c^The standard deviations of 10-time subsampling are indicated in parentheses.

^d^The sampling sites of paddy soils are given.

Concerning the iron oxides employed, 14, 16, 10, and 18 enrichment cultures came from incubations with goethite, lepidocrocite, hematite, and magnetite, respectively. Maximum microbial reduction rates by the iron reducers *Shewanella putrefaciens* and *Shewanella decolorationis* were reported in the order lepidocrocite ≥ goethite > hematite (Larsen and Postma, 2001; Bonneville et al., 2004, 2009; Li et al., 2012). In our experiments, the numbers of the obtained enrichment cultures decreased with decreasing the reported availability of iron oxides. According to the redox potentials (Weber et al., 2006), magnetite reduction coupled with organic compound oxidation represents the lowest energy production among the four iron oxides employed. However, the most numerous (i.e., 18) enrichments were generated with magnetite as electron acceptor. Magnetite is a mixed Fe(II)–Fe(III) mineral and exhibits electron conductivity, which can mediate electron syntrophy (Kato et al., 2010, 2012; Li et al., 2014) that might be profitable for the survival of microorganisms during the long-term enrichment process.

Concerning the source of the inoculum with which the enrichments were obtained, 40 out of 48 using paddy soils, 1 out of 16 using forest soil, 2 out of 16 using wetland soil, 9 out of 16 using ditch sediment, and 6 out of 40 using subseafloor sediments showed the existence of microorganisms. A large percentage (i.e., >80%) of incubation conditions for paddy soils could keep the microorganisms alive. Following methanogenesis, Fe(III) reduction is the second significant electron-accepting process in paddy soils (Yao et al., 1999), in which high activity and abundance of iron(III)-reducing bacteria have been found (Hori et al., 2007, 2010; Ding et al., 2015). This is a possible explanation for the high success rate of enrichment cultures from paddy soils associated with all the iron oxides. Enrichment cultures from ditch and subseafloor sediments were mostly acquired from incubations with magnetite and goethite, respectively, whereas those from wetland and forest soils were scarce and resulted from incubations with magnetite.

### Comparison of the Community Structures in Microbial Enrichment Cultures by Statistical Analyses of Illumina Sequencing and T-RFLP Data

Illumina sequencing of 16S rRNA gene amplicons determined the extent of microbial diversity in all 58 enrichment cultures obtained. A total of 1,845,969 sequences (i.e., a mean of 35,008 per library; maximum no., 88,924; minimum no., 5,484) of bacterial 16S rRNA genes were analyzed after removing the archaeal 16S rRNA gene sequences from original libraries (Supplementary Table S1). α-Diversity indices [i.e., Chao1, Shannon, and Simpson reciprocal (Shannon, 1948; Simpson, 1949; Chao, 1984)] were determined on the basis of equal numbers of bacterial sequences (n = 3,544; **Table 1**). The type of iron oxide had little effect on the microbial diversity indices, while differences in microbial diversity were observed among soil types. The richness index Chao1 of rice paddy soils represents a wide range of values (325–1,883). Neither the utilized basal medium nor the methanogen inhibitor BES influenced the variations. The richness tended to be positively correlated with the evenness given by the indices Shannon and Simpson reciprocal, which suggests that the paddy-soil enrichments consisted of high number of microbial species with equivalent relative abundances. Concerning the ditch-sediment enrichments, some of the microbial enrichments (IDs: e46, e33, and e45) represented low evenness, suggesting that particular microbial species dominated the enrichment cultures. The subseafloor-sediment cultures enriched with goethite mostly represented high values of Chao1 index. It has been reported that goethite was reduced considerably in subsurface sediments (Stucki et al., 2007; Komlos et al., 2008).

**FIGURE 2:**
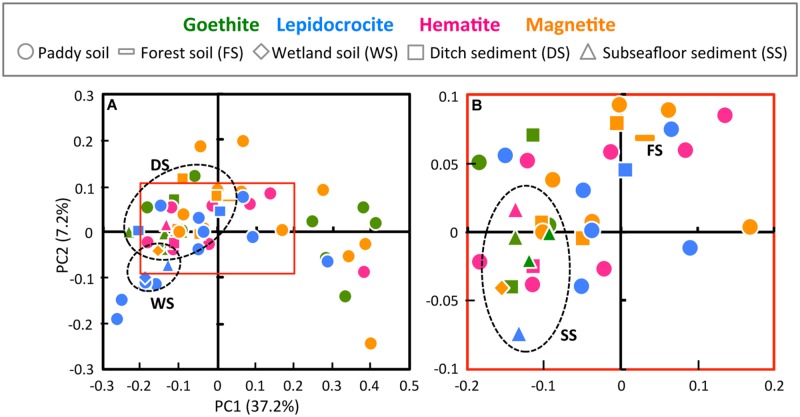
**Principal coordinate analysis (PCoA) plot of Illumina sequence data **(A)** and the high-resolution display of red square box in A (B).** PCoA plot was generated from the weighted UniFrac analysis of equal numbers of the bacterial sequences (*n* = 3,810). The x- and y-axes are indicated by the first and second coordinates, respectively, and the values in parentheses show the percentages of the community variation explained. Green, blue, pink, and orange symbols represent microbial enrichment cultures on goethite, lepidocrocite, hematite, and magnetite, respectively. Circles, bars, diamonds, squares, and triangles indicate enrichment cultures derived from paddy soil, forest soil, wetland soil, ditch sediment, and subseafloor sediment, respectively. Each type of soils and sediments other than paddy soil was circled by black dot line.

To compare the community structures in microbial enrichment cultures, the PCoA plot of Illumina sequence data was generated from the weighted UniFrac distance analysis using equal numbers of bacterial sequences (*n* = 3,810; **Figure 2**). In parallel, PCA of T-RFLP data with size and relative abundance of T-RFs was also conducted (Supplementary Figure S1). In the PCoA plot, a large part (i.e., 60.3%) of all the enrichments were located closely within the central area surrounded by red square (**Figure 2**). The degree of concentration at the center was remarkably high for enrichment cultures on hematite [i.e., 90% (9 out of 10 enrichments)] compared to those on the other iron oxides (50–61%). This indicates that hematite-enriched microbial communities were structurally similar to each other. Hematite was apparently highly selective for distinct microbial populations during the first cultivation. Lower bioavailability of iron oxides (Larsen and Postma, 2001; Bonneville et al., 2004, 2009; Li et al., 2012) and lower energy-productivity from redox coupling (Weber et al., 2006) might create higher selection pressures with hematite as substrate to limit microbial community members. Microbial enrichment cultures on four different forms of iron oxides (goethite, lepidocrocite, hematite, and magnetite) were overlapping to some extent. This was indicative of the existence of the core microorganisms involved in reduction of all the four iron(III) minerals. On the other hand, from a standpoint of inoculum source, the paddy-soil enrichments have scattered PCoA plots (**Figure 2**), which possibly reflected their large variations in the microbial diversity indices (**Table 1**). The other enrichment cultures were grouped according to the kind of soils and sediments (circled by black dot lines on the plots), indicating that the similar microbial community structures in enrichment cultures originated from the same soil type inoculated (**Figure 2**). Furthermore, from the methodological aspect, the results obtained from Illumina sequencing and T-RFLP had a good agreement in characterizing the community structure patterns. Nevertheless, it is noteworthy that PCoA of Illumina sequencing data differentiated more efficiently the microbial enrichments than PCA of T-RFLP data (**Figure 2** and Supplementary Figure S1).

### Comprehensive Identification of Constituent Members in Microbial Enrichment Cultures by Illumina Sequencing and Clone Library Analyses

**FIGURE 3:**
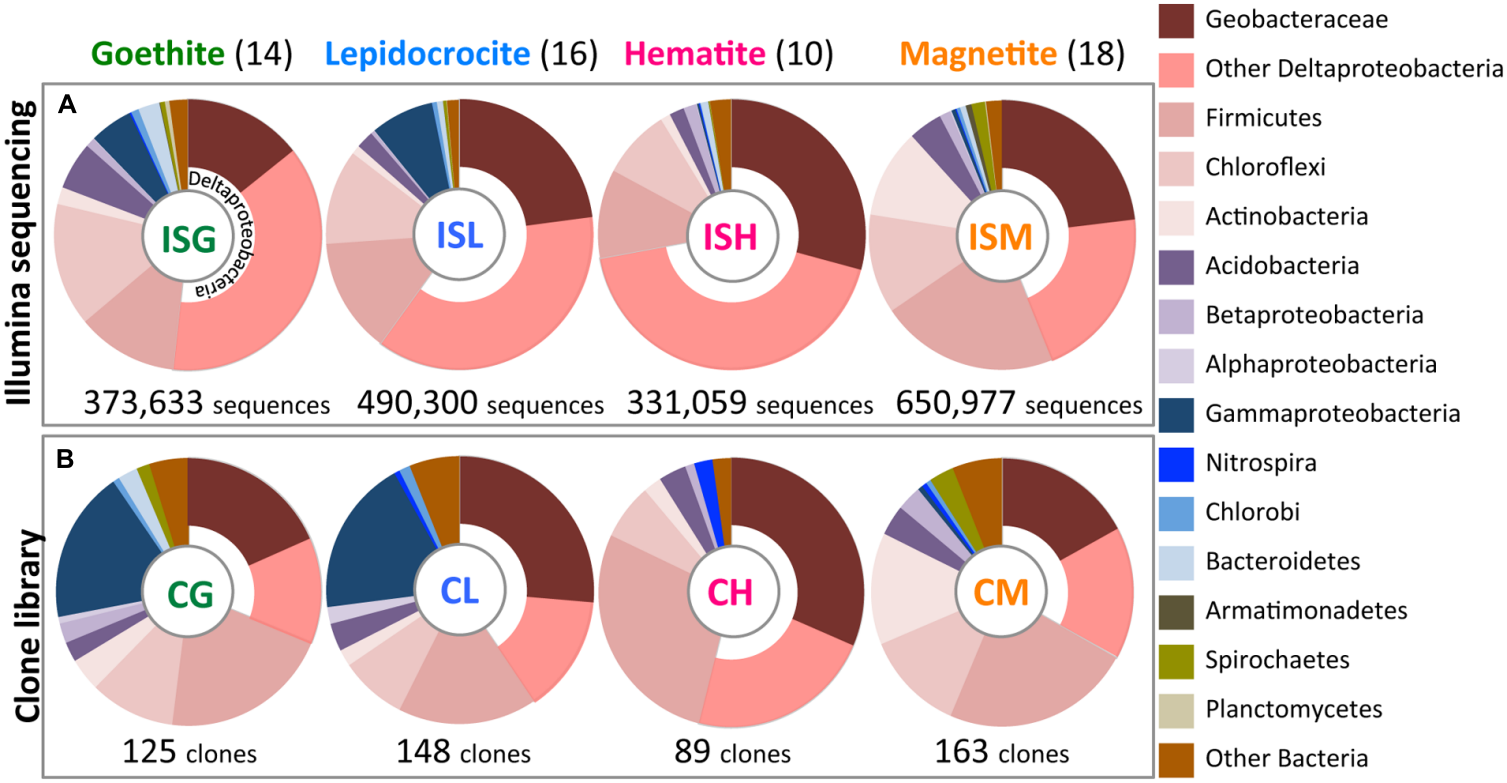
**Bacterial community structures in enrichment cultures as determined by Illumina sequencing **(A)** and clone library analysis (B).** Relative abundances of bacterial 16S rRNA gene sequences affiliated phylogenetically are shown in colors according to explanatory note on the right side. Illumina sequence (‘IS’) libraries from enrichment cultures on goethite (‘G’), lepidocrocite (‘L’), hematite (‘H’), and magnetite (‘M’) designated ISG, ISL, ISH, and ISM libraries, respectively. Clone (‘C’) libraries were defined in the same manner as CG, CL, CH, and CM libraries. Total numbers of sequences and clones obtained in this study are given below the circle graphs. Values in parentheses represent the numbers of the obtained enrichment cultures.

**FIGURE 4:**
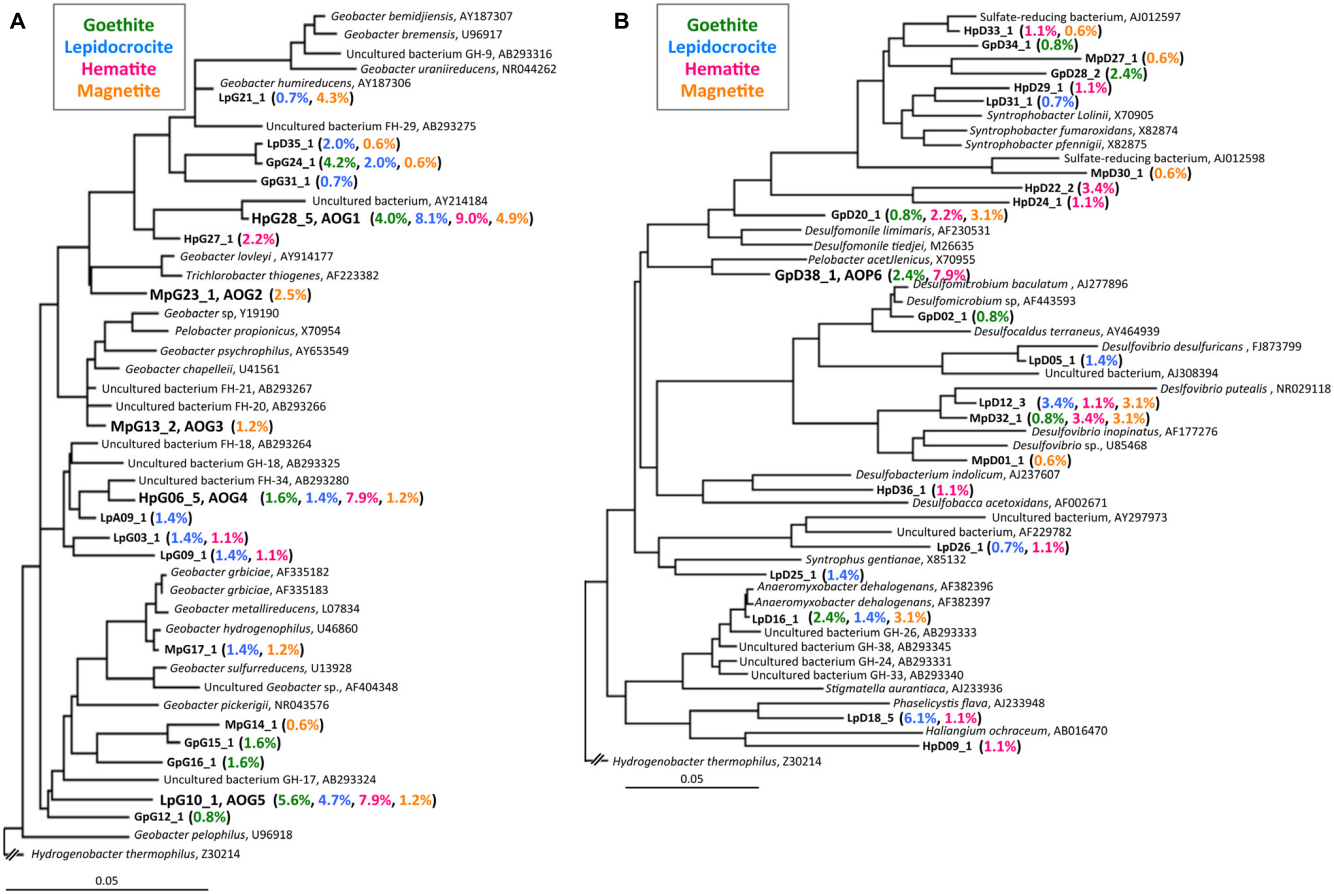
**Phylogenetic trees showing the relationships of 16S rRNA clone sequences related to the Geobacteraceae **(A)** and the other Deltaproteobacteria (B).** Clones obtained in this study are indicated in boldface and their relative abundances in the four clone libraries are given in parentheses (Green, blue, pink, and orange characters represent relative abundances in CG, CL, CH, and CM libraries, respectively). The scale bar represents 5% sequence divergence. GenBank accession numbers of reference sequences are indicated.

**FIGURE 5:**
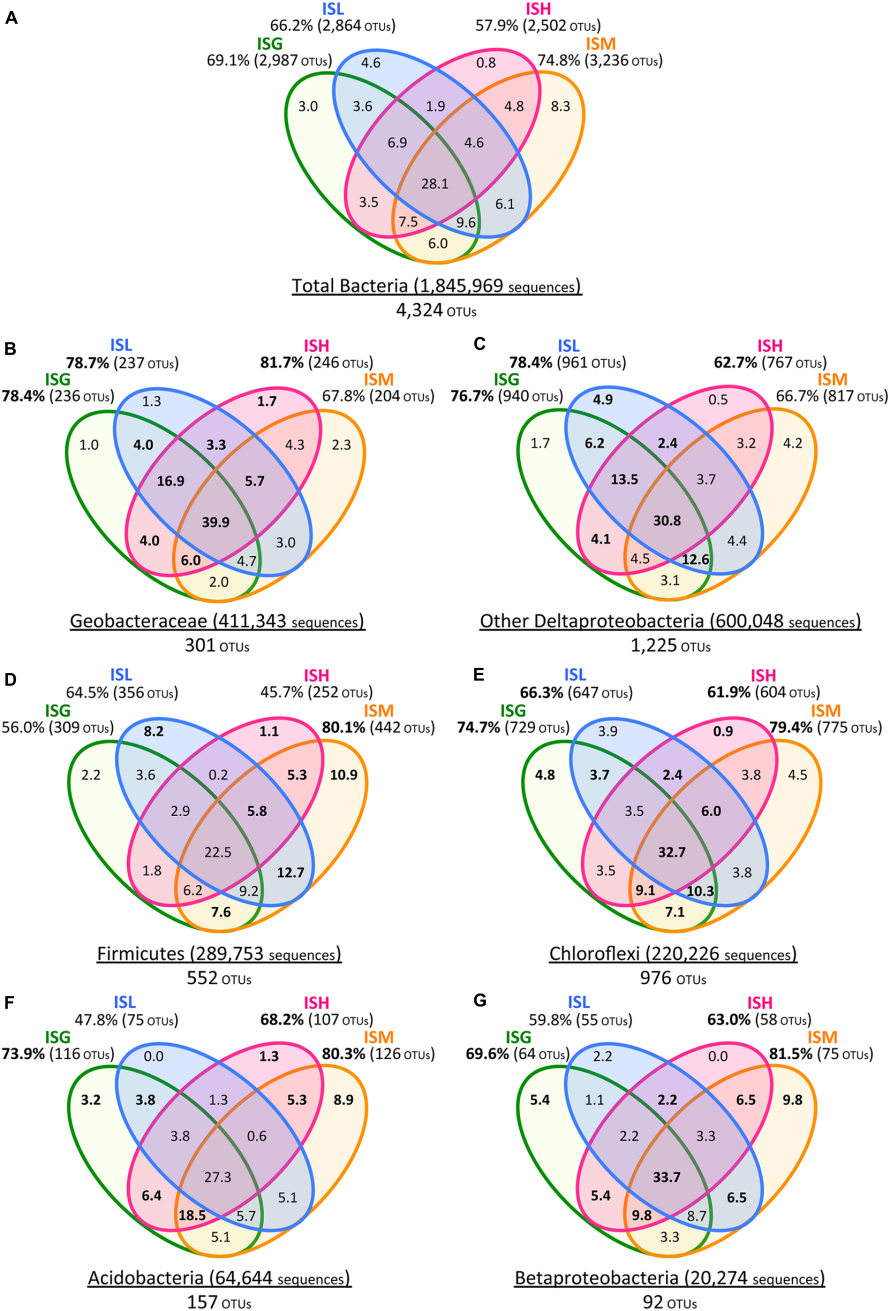
**Venn diagrams of Illumina sequence data for the total bacteria (A), Geobacteraceae (B), other Deltaproteobacteria (C), Firmicutes (D), Chloroflexi (E), Acidobacteria **(F)**, and Betaproteobacteria (G).** Green, blue, pink, and orange circles represent ISG, ISL, ISH, and ISM libraries, respectively. Total numbers of sequences and OTUs are given for each phylogenetic group. Values in Venn diagrams represent the percentages of OTUs to the total OTUs in each phylogenetic group. Relative frequencies of OTUs **(B–G)** that were higher than those of the total bacterial OTUs **(A)** are indicated in boldface.

A fine-scale phylogenetic characterization of the 58 enrichment cultures was carried out using Illumina sequencing and clone library analyses (**Figure 3**). PCoA indicated that microbial community structures in the paddy-soil enrichments encompassed those in the other soil- and sediment-enrichments (**Figure 2**). Thus, the molecular phylogenetic approaches were individualized for each iron oxide rather than for each soil type. Four Illumina sequence (‘IS’) libraries of bacterial 16S rRNA genes (the fragment lengths: ca. 250 bp) were constructed from microbial enrichment cultures on goethite (‘G’), lepidocrocite (‘L’), hematite (‘H’), and magnetite (‘M’): these designated the ISG library (n = 373,633), the ISL library (n = 490,300), the ISH library (n = 331,059), and the ISM (n = 650,977) library, respectively. In the same manner, four clone (‘C’) libraries of bacterial 16S rRNA genes (the fragment lengths: ca. 850 bp) were defined as the CG library (n = 125), the CL library (n = 148), the CH library (n = 89), and the CM library (n = 163). The IS libraries provided quantitative (i.e., relative-abundance) information of high accuracy concerning members in enrichment cultures because of the large amounts of 16S rRNA genes analyzed (Supplementary Table S1, **Figures 3A** and **5**). Complementarily, clone library analysis not only confirmed the results from Illumina sequencing but also produced the reliable qualitative (i.e., phylogenetic) information about the members owing to the long-fragment lengths of 16S rRNA genes sequenced (**Figures 3B** and **4**; Supplementary Figures S2A–D). A combination of high-throughput Illumina sequencing and conventional clone library analysis was a powerful approach to comprehensively identify constituent members in all the enrichment cultures with high resolution and high sensitivity.

The class Deltaproteobacteria highly dominated the four IS libraries, accounting for 51.7, 60.0, 72.1, and 43.8% to the total populations in ISG, ISL, ISH, and ISM libraries, respectively (**Figure 3A**). Within the Deltaproteobacteria, Geobacteraceae bacteria represented the highest relative abundance ranging from 14.1% in the ISG library to 29.1% in the ISH library. The predominance of the Deltaproteobacteria that mainly consisted of Geobacteraceae bacteria was also confirmed by clone library analysis (**Figure 3B**). Phylogenetic trees of the Geobacteraceae and the other Deltaproteobacteria revealed that most of the clones obtained were affiliated within novel lineages of *Geobacter* spp. (**Figure 4A**), *Pelobacter* spp., *Desulfovibrio* spp., and *Anaeromyxobacter* spp. (**Figure 4B**) that are all known to be dissimilatory iron(III) reducers (Bale et al., 1997; He and Sanford, 2003; Lovley et al., 2004, 2011). Furthermore, there were clones related to sulfate-reducing and/or syntrophic bacteria such as *Syntrophobacter* spp. and *Syntrophus* spp. They might be directly engaged in iron reduction, but as another scenario, they possibly catalyzed syntrophic oxidation of acetate and/or the other volatile fatty acids in partnership with methanogens in the absence of BES (Hattori, 2008; McInerney et al., 2008; Hori et al., 2011; Gan et al., 2012).

With a special focus on OTU-level distribution in the IS libraries, 411,343 sequences and 600,048 sequences out of the total 1,845,969 sequences were assigned to 301 OTUs within the Geobacteraceae and to 1,225 OTUs within the other Deltaproteobacteria, respectively (**Figures 5A–C**). Venn diagrams of the IS libraries elucidated that the core OTUs enriched with all the four iron oxides were predominant in the Geobacteraceae, accounting for a relative frequency of 39.9% (**Figure 5B**) that is much higher than that in the total bacteria (i.e., 28.1%; **Figure 5A**). This implies that the microorganisms involved in the reduction of all the four iron oxides were foremost members within the Geobacteraceae. In addition, relative frequencies of the Geobacteraceae OTUs stimulated by hematite [ISH library (81.7%; 246/301 OTUs) in **Figure 5B**] were greater than those of the total bacterial OTUs enriched with hematite [ISH library (57.9%; 2,502/4,324 OTUs) in **Figure 5A**]. With respect to the other Deltaproteobacteria, the higher relative frequencies of OTUs were found in enrichment cultures on lepidocrocite [ISL library (78.4%; 961/1,225 OTUs) in **Figure 5C** versus ISL library (66.2%; 2,864/4,324 OTUs) in **Figure 5A**]. Accordingly, for the Geobacteraceae and the other Deltaproteobacteria, the higher relative frequencies of OTUs were apparent in the ISG, ISL, and ISH libraries (**Figures 5B,C**) as compared to those of the total bacterial OTUs in these libraries (**Figure 5A**). This suggests that the Deltaproteobacteria populations mainly contributed to the reduction of goethite, lepidocrocite, and hematite in comparison with other taxonomic groups; more specifically, the Geobacteraceae and the other Deltaproteobacteria had relatively high abilities to reduce hematite and lepidocrocite, respectively.

The phyla Firmicutes and Chloroflexi were the next dominant sequences in all the IS libraries, occupying 10.6–21.7% and 8.4–14.8% of the total populations, respectively (**Figure 3A**), which is in line with the results from clone library analysis (**Figure 3B**). Venn diagrams of the IS libraries showed metabolic activation of these phylogenetic groups according to the types of iron oxides supplemented (**Figures 5A,D,E**). The Firmicutes OTUs had higher relative frequencies in enrichment cultures particularly with magnetite [ISM library (80.1%; 442/552 OTUs) in **Figure 5D**] than the total bacterial OTUs [ISM library (74.8%; 3,236/4,324 OTUs) in **Figure 5A**]. The slightly higher frequencies of the Chloroflexi OTUs were found broadly in all the IS libraries (ISG: 74.7%, ISL: 66.3%, ISH: 61.9%, and ISM: 79.4% in **Figure 5E** versus those percentages in **Figure 5A**). In phylogenetic trees of the Firmicutes and the Chloroflexi (Supplementary Figures S2A,B), some of the retrieved clones were related to the known carbohydrate-degrading fermentative bacteria (Collins et al., 1994; Sekiguchi et al., 2003). In combination with the results from Venn diagrams (**Figure 5**), magnetite for the Firmicutes and all the crystalline iron oxides for the Chloroflexi might have served as sinks for excess electrons generated from fermentative degradation of organic compounds as reported previously (Lovley and Phillips, 1986b; Dobbin et al., 1999; Lentini et al., 2012). Yet recent studies reported that some bacteria classified within these phyla could transfer electrons directly or indirectly to ferric iron oxides for energy conservation (Wrighton et al., 2011; Kawaichi et al., 2013). It is thus likely that Firmicutes and Chloroflexi enriched in this study catalyzed dissimilatory reduction of crystalline iron(III) oxides.

Meanwhile, the phylum Acidobacteria and the class Betaproteobacteria existed as relatively minor constituents in the IS libraries (i.e., Acidobacteria: 1.8–5.7%, Betaproteobacteria: 0.2–1.6%) as also supported by clone library data (**Figure 3**). Clones related to the known iron reducer *Geothrix fermentans* (Nevin and Lovley, 2002) and the putative goethite reducers (i.e., the uncultured bacteria GH-48 and GH-47) we have previously identified using RNA-SIP (Hori et al., 2010) were found in the phylogenetic trees (Supplementary Figures S2C,D). Venn diagrams of the IS libraries suggested the preferential involvement of the Acidobacteria and the Betaproteobacteria in the reduction of goethite, hematite, and magnetite but not lepidocrocite (**Figures 5F,G**).

### Isolation of Novel Iron Reducers from Microbial Enrichment Cultures and their Physiological Characters

**FIGURE 6:**
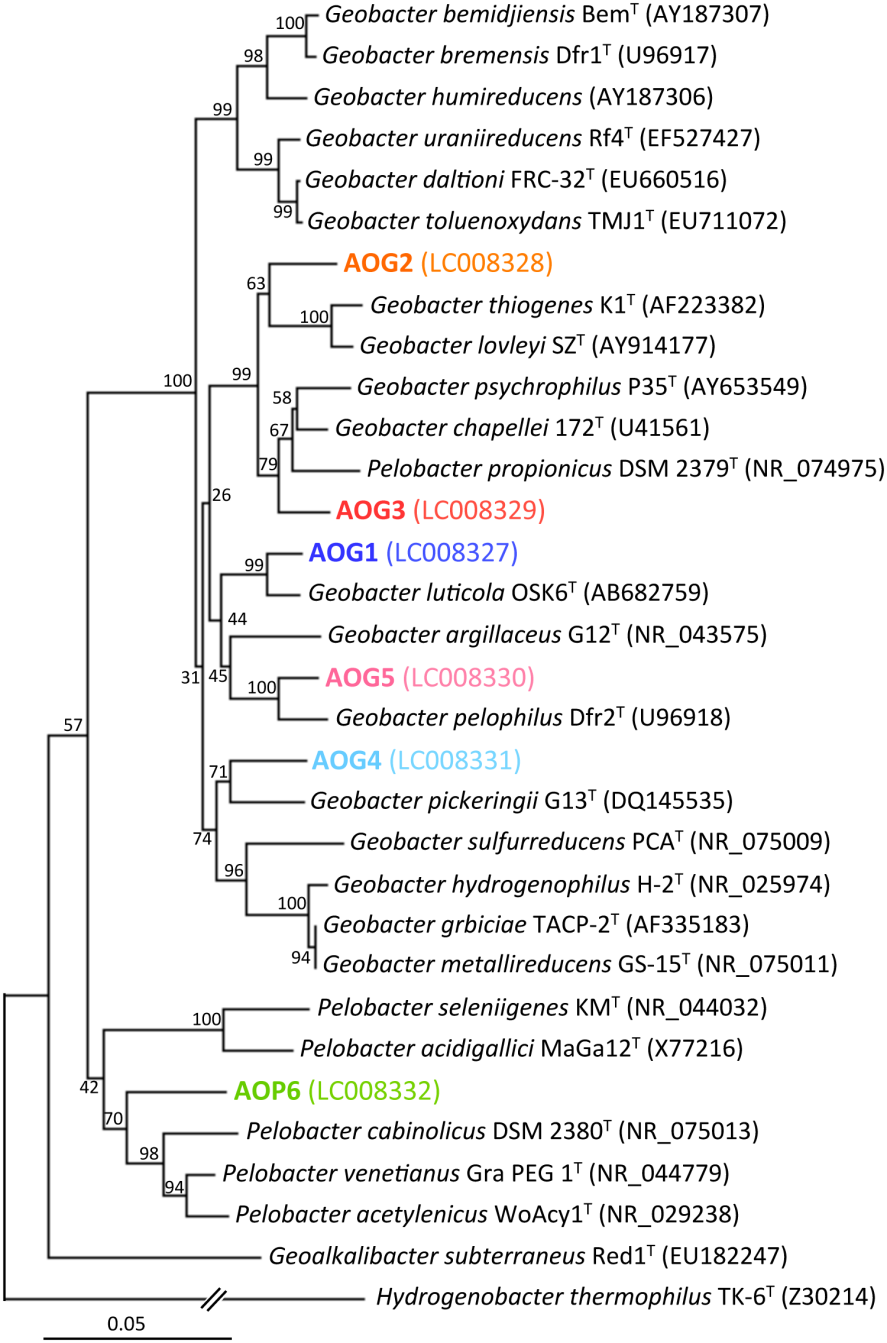
**Phylogenetic tree showing the relationship of 16S rRNA genes from isolates (the strains AOG1–5 and AOP6) and their known cultured relatives.** The aligned nearly full-lengths of 16S rRNA genes were utilized for the tree construction using the neighbor-joining method. Bootstrap values were obtained from 1,000 replications. The scale bar represents 5% sequence divergence. GenBank accession numbers of sequences are given in parentheses.

After the first cultivation, 38 out of the total 58 enrichment cultures were selected on the basis of the accumulation of phylogenetically novel iron reducers. The selected enrichment cultures were transferred to fresh soluble-Fe(III) media to proliferate the enriched novel iron(III)-reducing bacteria. Through extinction dilution culture and/or single colony isolation, six strains (named as AOG1–5 and AOP6) within the Deltaproteobacteria were eventually obtained. According to the nearly full-lengths of 16S rRNA gene sequence determined, the five strains AOG1–5 belonged to the genus *Geobacter* and the strain AOP6 to *Pelobacter* (**Figure 6**). In detail, the strains AOG1 and AOG4 (the clones HpG28_5 and HpG06_5 in **Figure 4A**) were isolated from paddy soils in Hokkaido and Italy using lepidocrocite as electron acceptor. They were related to the recently isolated *Geobacter luticola* (AB682759; 98.1% sequence similarity; Viulu et al., 2013) and the kaolin-clay derived *G. pickeringii* (NR_043576; 95.5% sequence similarity; Shelobolina et al., 2007), respectively. The strains AOG2 and AOG3 (the clones MpG23_1 and MpG13_2) were obtained from wetland and forest soils supplemented with magnetite and they had close phylogenetic relationships with the metal-reducing and dehalo-respiring *G. lovleyi* (NR_074979; 96.4% sequence similarity; Sung et al., 2006) and the subsurface-aquifer originated *G.chapelleii* (NR_025982; 96.7% sequence similaity; Coates et al., 1996), respectively. The strain AOG5 (clone: LpG10_1) isolated from the agricultural ditch sediment with supplementation of hematite was related to *G. pickeringii* (NR_026077; 94.8% sequence similarity; Straub et al., 1998). The strain AOP6 (clone: GpD38_1) was obtained from the subseafloor sediment added with goethite and it was related to *Pelobacter acetylenicus* (NR_029238; 95.5% sequence similarity; Evers et al., 1993). Because isolation of these microorganisms stemmed from all the different inoculum sources (i.e., paddy soil, forest soil, wetland soil, agricultural ditch sediment, or subseafloor sediment) and different iron(III) oxides (i.e., goethite, lepidocrocite, hematite, or magnetite), a variety of incubation conditions employed for the primary enrichment were effective in obtaining these novel microorganisms in pure cultures. Taken together, the two-stage cultivation method successfully isolated novel iron reducers that had low 16S rRNA sequence similarities (i.e., 94.8–98.1%) to their known cultured relatives.

**FIGURE 7:**
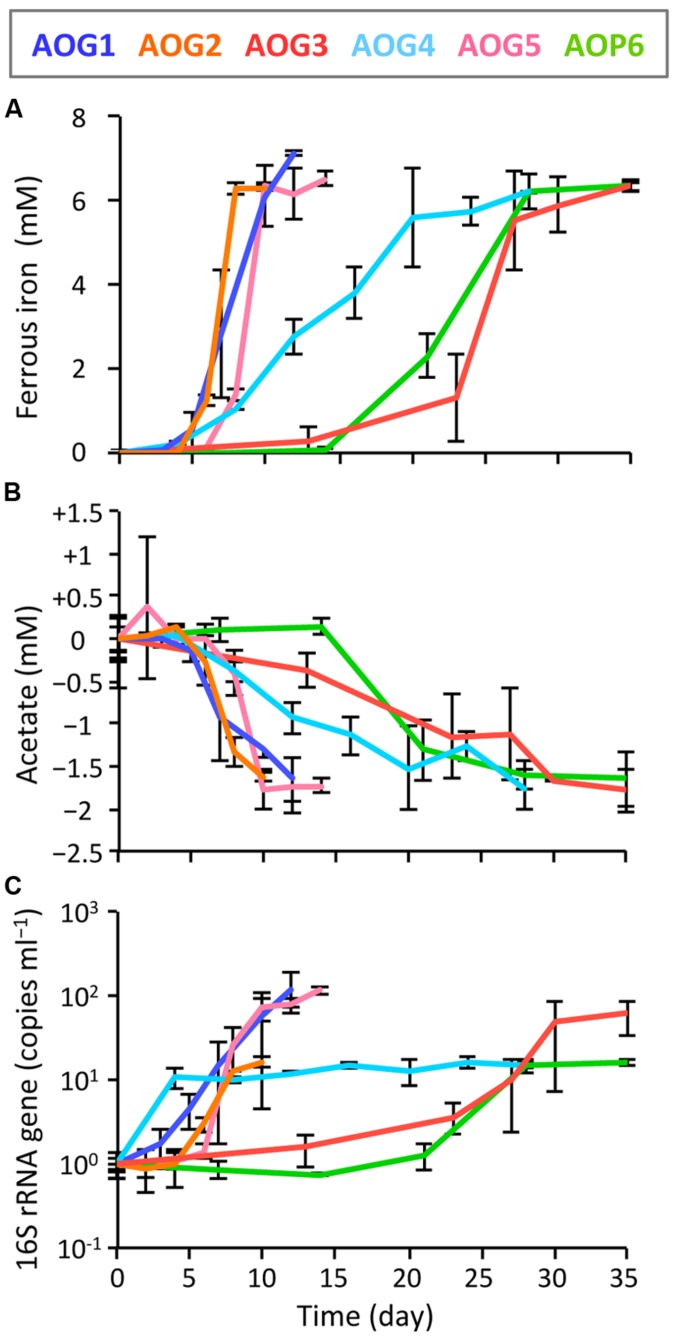
**Kinetics of physiological parameters during the incubation of isolates, i.e., the strains AOG1–5 and AOP6; **(A)** ferrous iron, **(B)** acetate, and **(C)** increase in copy number of 16S rRNA genes.** Fe(III)-NTA and acetate were used as electron acceptor and donor, respectively. Each colored line represents the parameter from one strain: the correspondence between colors and strains is shown in the explanatory note. The error bars indicate the standard deviations of three replications.

In order to clarify whether or not these isolates were truly dissimilatory ferric iron reducers, physiological parameters [i.e., Fe(III) reduction, acetate degradation, cell growth, and protein synthesis] were monitored during the incubation of isolates supplemented with Fe(III)-NTA as electron acceptor and acetate as electron donor (**Figure 7**). The strains AOG1, AOG2, and AOG5 rapidly reduced ferric iron; concentrations of ferrous iron increased with incubation time reaching a plateau with final values of 6.84–7.14 mM within ∼10 days (**Figure 7A**). The other three strains showed relatively slow iron-reduction rates. It took ∼30 days to reach a maximum Fe(II) concentration (6.88–7.32 mM). Degradation of acetate coincided with iron(III) reduction (**Figure 7B**). The found redox coupling of iron reduction and acetate oxidation roughly matched with the expected stoichiometry [acetate:iron(III) = 1:8], although the amounts of the acetate degraded were somewhat higher. This might be due to the acetate utilization by microorganisms not only for dissimilation (electron donor) but also for assimilation (carbon source). The cell growth was evaluated by quantifying the copy numbers of 16S rRNA genes in the cultures (**Figure 7C**). The growth rates of six strains were different from each other; the strains AOG1 and AOG5 exhibited a two-order increase in their 16S rRNA genes (1.17–1.22 × 10^2^ copy increase ml^-1^) and the others increased by one-order of magnitude (1.52–6.07 × 10 copy increase ml^-1^). Proliferation of microbial cells (**Figure 7C**) and synthesis of proteins (data not shown) were mostly synchronized with the oxidation–reduction reaction except those of the strain AOG4. Although the strain AOG4 grew at the beginning of the incubation and afterward remained at the same population level, they vigorously expressed protein after day 15 during which iron reduction occurred. Consequently, these results indicate that all the isolates were able to grow on acetate and ferric iron but their physiological characteristics differed considerably in terms of growth rate.

## Conclusion

Due to the low solubility and high crystallinity being linked to low bioavailability (Bonneville et al., 2009; Cutting et al., 2009), natural occurrence of microbial reduction of crystalline iron(III) oxides is largely unknown while iron-reducing microorganisms have been extensively investigated by using solubilized iron(III) oxide (Lovley et al., 2004). In order to better understand microorganisms that contribute to the reduction of crystallized ferric iron, we primarily enriched soil and sediment microbial communities with acetate and crystalline iron oxide, i.e., goethite, lepidocrocite, hematite, or magnetite, followed by isolation of target microorganisms using readily utilizable ferric iron that facilitates effective isolation, referred to as two-stage cultivation in this study. By employing high-throughput Illumina sequencing, T-RFLP, and clone library analyses, we successfully selected enrichment cultures that harbored bacteria presumably responsible for reduction of crystalline irons instead of canonical metabolic analysis (i.e., ferrous iron formation), suggesting that the strategy we have taken is a very useful approach for further purification and isolation of slowly growing microorganisms, which would not otherwise be accessible. The two-stage cultivation method successfully isolated six novel iron(III)-reducing bacteria belonging to the genera *Geobacter* and *Pelobacter*. Although the ability of these isolates to reduce crystallized ferric irons has yet to be verified, their involvement in the reduction of crystalline iron oxides in natural environments is quite likely. The accessibility and metabolic responses of these isolates to highly crystallized irons should be investigated to clarify the true reduction of the Fe(III) oxides and the mechanisms underlying.

## Conflict of Interest Statement

The authors declare that the research was conducted in the absence of any commercial or financial relationships that could be construed as a potential conflict of interest.

## Footnotes

1 http://www.arb-home.de

2 http://www.ddbj.nig.ac.jp

3 http://metagenomics.anl.gov/
