# Supplementary material for: Isolation of microorganisms involved in reduction of crystalline iron(III) oxides in natural environments
Source: Front Microbiol. 2015 May 5;6:386. doi: 10.3389/fmicb.2015.00386 (PMC4419728; doi:10.3389/fmicb.2015.00386)
Supplement: Supplementary file 1 [file Data_Sheet_1.PDF]

Supplementary Material

Isolation of microorganisms involved in reduction of crystalline iron(III) oxides in natural environments

Tomoyuki Hori[1†]*, Tomo Aoyagi[1†], Hideomi Itoh[2], Takashi Narihiro[3], Azusa Oikawa[2‡], Kiyofumi Suzuki[4], Atsushi Ogata[1], Michael W. Friedrich[5], Ralf Conrad[6], and Yoichi Kamagata[3]

[1] Environmental Management Research Institute, National Institute of Advanced Industrial Science and Technology (AIST), Tsukuba, Japan
[2] Bioproduction Research Institute, National Institute of Advanced Industrial Science and Technology (AIST), Sapporo, Japan
[3] Bioproduction Research Institute, National Institute of Advanced Industrial Science and Technology (AIST), Tsukuba, Japan
[4] Methane Hydrate Research & Development Division, Japan Oil, Gas and Metals National Corporation (JOGMEC), Chiba, Japan
[5] Microbial Ecophysiology group, Faculty of Biology/Chemistry and MARUM, University of Bremen, Bremen, Germany
[6] Max Planck Institute for Terrestrial Microbiology, Marburg, Germany

[†]***T. Hori and T. Aoyagi contributed equally to this work***

[‡]**Present address:** Department of biotechnology, Hokkaido College of High Technology, Eniwa, Japan

***Correspondence:** Tomoyuki Hori, Environmental Management Research Institute, National Institute of Advanced Industrial Science and Technology (AIST), Onogawa 16-1, Tsukuba, Ibaraki 305-8569, Japan.
e-mail: hori-tomo@aist.go.jp

Keywords: Isolation, iron-reducing bacteria, crystalline iron(III) oxide, *Geobacter*, high-throughput sequencing.

Supplementary Figures and Tables

Figure S1 | Principal component analysis (PCA) plot of T-RFLP data (A) and the high-resolution display of red square box in A (B). PCA plot was based on size and relative abundance of T-RFs. Green, blue, pink, and orange symbols represent microbial enrichment cultures on goethite, lepidocrocite, hematite, and magnetite, respectively. Circles, bars, diamonds, squares, and triangles indicate enrichment cultures derived from paddy soil, forest soil, wetland soil, ditch sediment, and subseafloor sediment, respectively. Each type of soils and sediments other than paddy soil was circled by black dot line.

Figure S2 | Phylogenetic trees showing the relationships of 16S rRNA clone sequences related to the Firmicutes (A), Chloroflexi (B), Acidobacteria (C), and Betaproteobacteria (D). Clones obtained in this study are indicated in boldface and their relative abundances in the four clone libraries are given in parentheses (Green, blue, pink, and orange characters represent relative

abundances in CG, CL, CH, and CM libraries, respectively). The scale bar represents 5% sequence divergence. GenBank accession numbers of reference sequences are indicated.

Table S1 | Summary of Illumina sequence data.

Hori et al., Figure S1

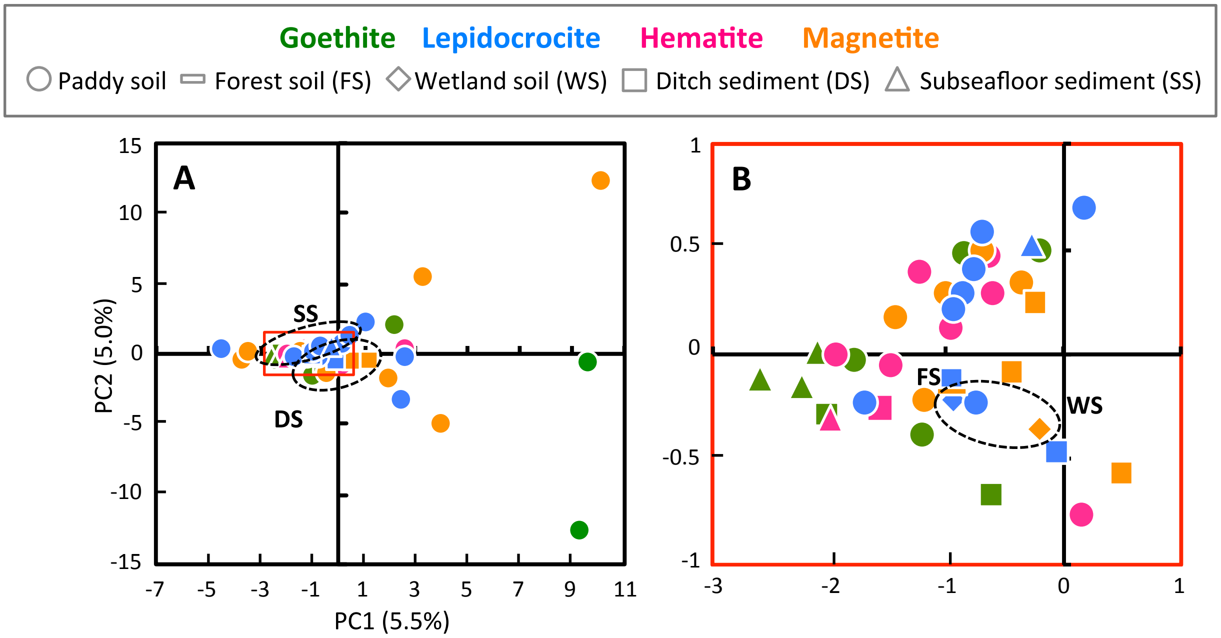

Hori et al., Figure S2A

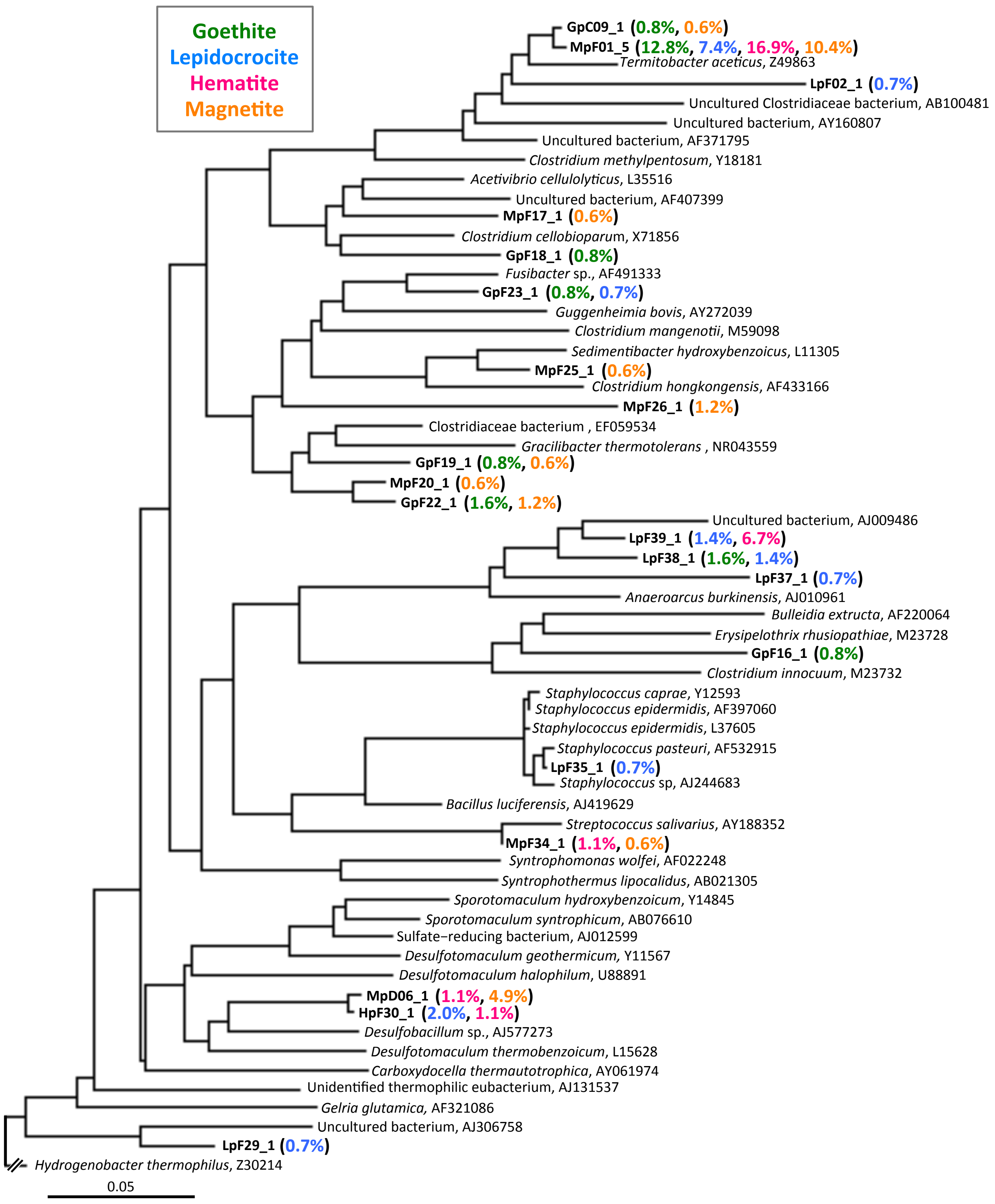

Hori et al., Figure S2B

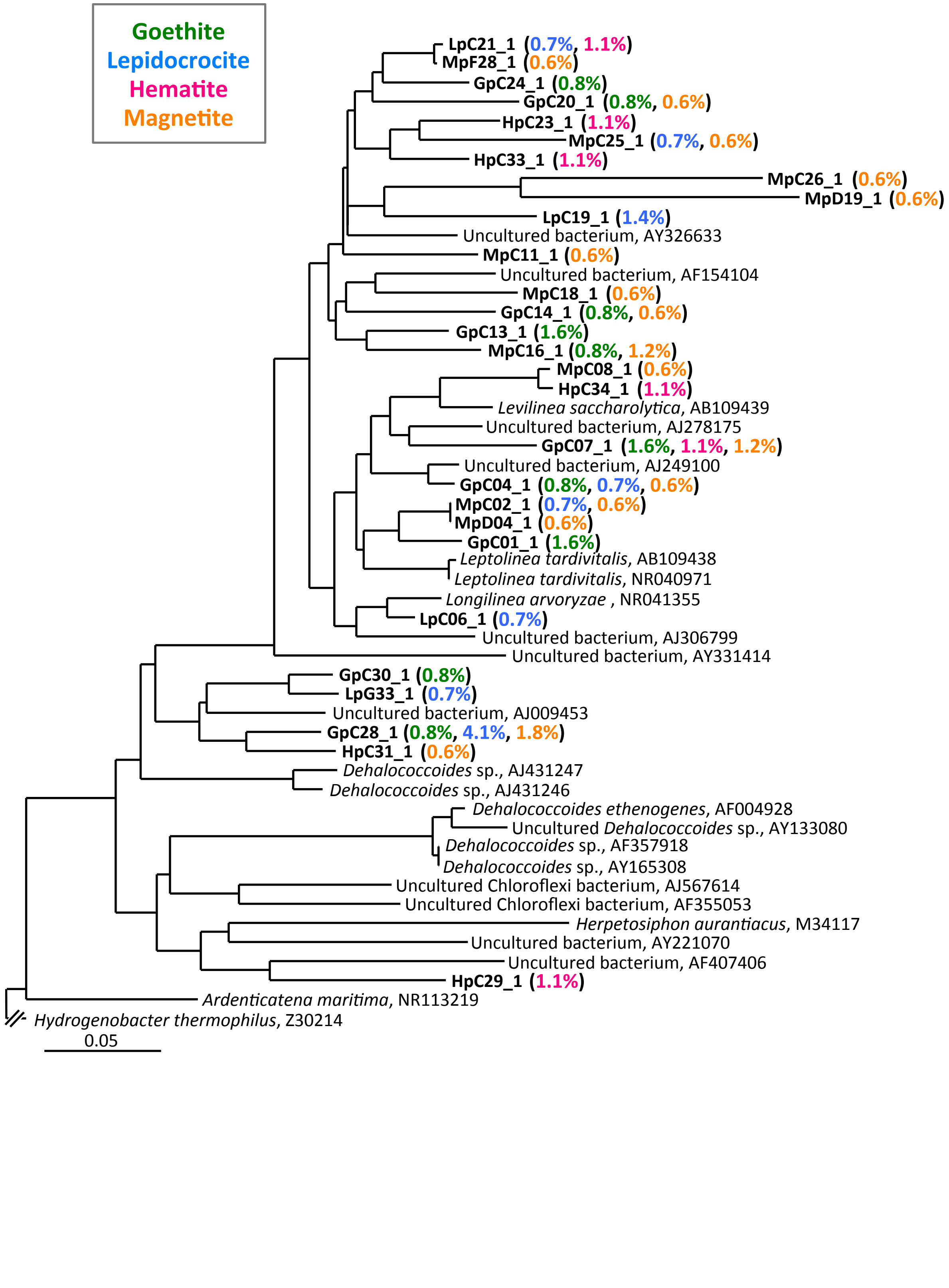

Hori et al., Figure S2C

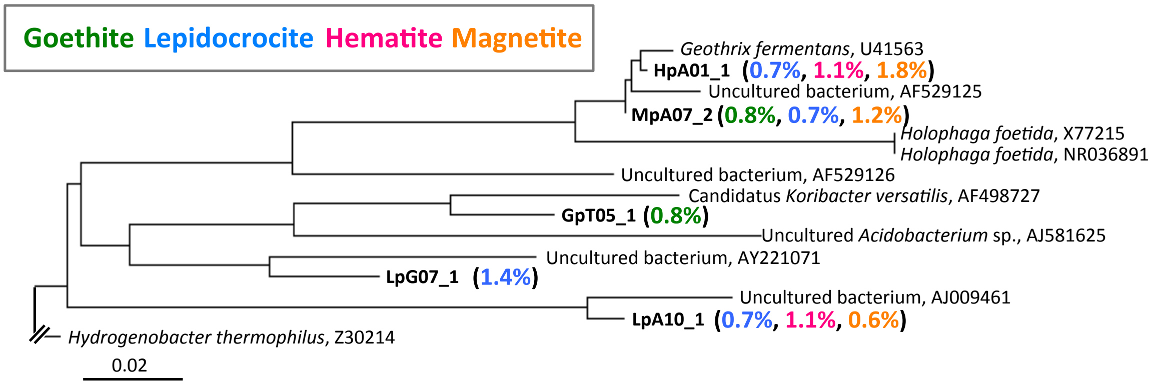

Hori et al., Figure S2D

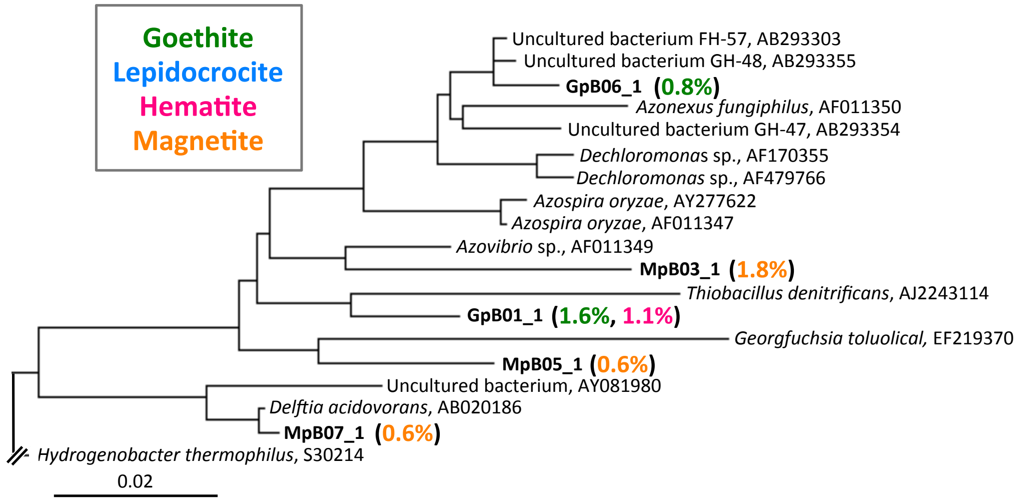

Hori et al., Table S1

MG-RAST ID	Library ID	No. of total sequences	No. of bacterial sequences
4589480.3	e05	19,306	18,504
4589486.3	e11	37,632	36,720
4589522.3	e50	19,426	17,168
4589508.3	e35	42,142	41,487
4589500.3	e27	7,986	7,537
4589488.3	e14	59,041	55,374
4589512.3	e39	28,427	26,435
4589492.3	e18	93,484	88,924
4589481.3	e06	31,783	30,227
4589485.3	e10	12,734	11,714
4589523.3	e51	47,987	43,849
4589509.3	e36	63,006	55,886
4589501.3	e28	47,303	44,835
4589489.3	e15	16,860	16,261
4589526.3	e54	38,069	36,538
4589513.3	e40	39,553	33,891
4589504.3	e31	9,345	8,860
4589528.3	e56	8,743	7,921
4589493.3	e19	32,977	27,852
4589516.3	e43	15,604	13,839
4589484.3	e09	5,764	5,484
4589521.3	e49	37,257	36,338
4589487.3	e13	40,451	36,915
4589525.3	e53	41,226	39,878
4589511.3	e38	19,023	18,559
4589503.3	e30	37,751	35,797
4589491.3	e17	26,144	24,498
4589515.3	e42	52,868	50,714
4589482.3	e07	59,321	57,470
4589483.3	e08	9,172	8,527
4589524.3	e52	49,855	49,251
4589510.3	e37	60,734	57,215
4589502.3	e29	25,060	23,910
4589490.3	e16	20,622	20,146
4589527.3	e55	46,433	43,944
4589514.3	e41	31,185	30,132
4589505.3	e32	42,307	41,395
4610839.3	e20	45,633	44,023
4589529.3	e57	71,915	71,445
4589517.3	e44	37,799	36,869
4589497.3	e24	42,843	41,857
4589519.3	e46	39,370	38,778
4589506.3	e33	70,720	69,455
4589498.3	e25	43,050	42,023
4589518.3	e45	70,216	68,941
4589507.3	e34	58,018	57,386
4589499.3	e26	67,364	62,098
4589530.3	e59	37,010	36,715
4589520.3	e48	50,873	50,217
4589535.3	e65	14,686	13,693
4589536.3	e66	13,550	13,046
4589533.3	e62	16,610	15,178
4589531.3	e60	66,904	62,086
4589532.3	e61	53,258	48,935
4589534.3	e64	30,928	30,511
4589495.3	e21	8,202	7,739
4589496.3	e22	9,764	9,482
4589479.3	e01	6,413	5,965
